# Evaluation of the Hippocampal Normal Tissue Complication Model in a Prospective Cohort of Low Grade Glioma Patients—An Analysis Within the EORTC 22033 Clinical Trial

**DOI:** 10.3389/fonc.2019.00991

**Published:** 2019-10-01

**Authors:** Jaap Jaspers, Alejandra Mèndez Romero, Mischa S. Hoogeman, Martin van den Bent, Ruud G. J. Wiggenraad, Martin J. B. Taphoorn, Danielle B. P. Eekers, Frank J. Lagerwaard, Anna Maria Lucas Calduch, Brigitta G. Baumert, Martin Klein

**Affiliations:** ^1^Department of Radiation Oncology, Erasmus Medical Center Cancer Institute, Rotterdam, Netherlands; ^2^Department of Neurology, Erasmus Medical Center, Rotterdam, Netherlands; ^3^Department of Radiation Oncology, Haaglanden Medical Center, Leidschendam, Netherlands; ^4^Department of Neurology, Haaglanden Medical Center, The Hague, Netherlands; ^5^Department of Radiotherapy, School for Oncology and Developmental Biology, Maastricht University, Maastricht, Netherlands; ^6^Department of Radiation Oncology, Medical Center, VU University Amsterdam, Amsterdam, Netherlands; ^7^Department of Radiation Oncology, Catalan Institute of Oncology, Barcelona, Spain; ^8^Department of Radiation Oncology, University Hospital Bonn, Bonn, Germany; ^9^Department of Medical Psychology, University Medical Center Amsterdam, Amsterdam, Netherlands

**Keywords:** NTCP (normal tissue complication probability) model, low grade glioma (LGG), model verification and validation, neurocognition, memory, late effect of cancer treatment, radiotherapy—adverse effects

## Abstract

**Purpose:** To evaluate the performance of the hippocampal normal tissue complication model that relates dose to the bilateral hippocampus to memory impairment at 18 months post-treatment in a population of low-grade glioma (LGG) patients.

**Methods:** LGG patients treated within the radiotherapy-only arm of the EORTC 22033-26033 trial were analyzed. Hippocampal dose parameters were calculated from the original radiotherapy plans. Difference in Rey Verbal Auditory Learning test delayed recall (AVLT-DR) performance pre-and 18 (±4) months post-treatment was compared to reference data from the Maastricht Aging study. The NTCP model published by Gondi et al. was applied to the dosimetric data and model predictions were compared to actual neurocognitive outcome.

**Results:** A total of 29 patients met inclusion criteria. Mean dose in EQD2 Gy to the bilateral hippocampus was 39.8 Gy (95% CI 34.3–44.4 Gy), the median dose to 40% of the bilateral hippocampus was 47.2 EQD2 Gy. The model predicted a risk of memory impairment exceeding 99% in 22 patients. However, only seven patients were found to have a significant decline in AVLT-dr score.

**Conclusions:** In this dataset of only LGG patients treated with radiotherapy the hippocampus NTCP model did not perform as expected to predict cognitive decline based on dose to 40% of the bilateral hippocampus. Caution should be taken when extrapolating this model outside of the range of dose-volume parameters in which it was developed.

## Introduction

Low grade glioma (LGG) are a group of relatively slow growing primary brain neoplasms, mainly occurring in those between 30 and 50 years of age (1, 2). Modern treatment for LGG patients comprises surgery followed by radiotherapy and adjuvant chemotherapy (3). Overall survival was recently reported to be 13.3 years (4), but can vary with molecular subtype.

With many LGG patients living for many years or even decades after treatment, the late adverse effects of treatment on quality of life and neurocognitive functioning are of increasing importance. Although both the tumor itself, as well as the use of anticonvulsant therapy, have a deleterious effect on neurocognitive function (5, 6), radiotherapy (RT) in particular has been associated with a negative impact on neurocognitive function. This late effect of radiotherapy was found in several series with a longer follow-up (7, 8), however, it was not found in several studies that limited observation to the first 5 years (9–12).

A dose response relationship with decreasing neurocognitive performance (specifically memory) has been attributed to the hippocampal area (13). A NTCP model for memory impairment was proposed by Gondi et al. (14). In this study, 18 patients undergoing fractionated stereotactic radiotherapy for benign and low-grade tumors (9 vestibular schwannomas, 2 pituitary adenomas, 3 meningiomas, and 4 low grade gliomas) completed a comprehensive baseline neurocognitive assessment and a repeat assessment at 18 months. A control group of 6 non-irradiated subjects was tested as well, allowing for the use of Z scores for performance change. Dose in excess of 7.3 EQD2 Gy to 40% of bilateral hippocampus were found to be significantly correlated to a decrease in Wechsler Memory Scale III–Word Lists delayed recall score, a test that measures verbal memory performance.

Although this model is routinely used in the clinic, its performance has not yet been quantified in the setting of partial brain irradiation in a population of LGG patients. We analyzed data from a recently completed and published randomized phase III trial, where LGG patients in the control arm were treated exclusively with focal radiotherapy up to 50.4 Gy (15) and compared the predicted risk of neuropsychological impairment with the actual outcome.

## Materials and Methods

### Patient Population

Data was acquired within the EORTC 22033-26033 (NCT00182819) trial, which is a prospective, randomized, open-label, phase 3 Intergroup study (European Organisation for Research and Treatment of Cancer [EORTC] Radiotherapy and Brain Tumor Groups, National Cancer Institute of Canada [NCIC] Clinical Trials Group, Trans Tasman Radiation Oncology Group [TROG], Medical Research Council [MRC] Clinical Trials Unit). The study was approved by the institutional review boards and ethics committees of all participating centers. All patients provided written informed consent at the time of registration (15).

In the aforementioned trial, patients of 18 years of age or older with histologically confirmed and centrally reviewed low-grade (WHO 2) glioma (diffuse astrocytoma, oligoastrocytoma and oligodendroglioma, WHO classification 2006) with at least one high-risk feature (age >40 years, progressive disease, tumor size >5 cm, tumor crossing midline, any focal neurological deficit) were randomly assigned to treatment with either radiotherapy (28 × 1.8 Gy) or temozolomide chemotherapy. Between September 2005 and March 2010 477 patients were randomized. The study design, treatment details and the results of the primary analysis have been described elsewhere (15). A total of 103 patients from preselected medical centers also underwent a detailed neurocognitive examination consisting of the Rey Auditory Verbal Learning test (AVLT), Concept Shifting test, Categoric Word Fluency test, and the Digit-Symbol Substitution test. Neurocognitive tests were conducted at randomization and then every 6 months until to tumor progression or death.

The analysis presented herein contains patients with retrievable radiotherapy planning data and neuropsychological testing at both baseline and 18 (±4 months). The neurocognitive analysis for the entire patient population of EORTC 22033-26033 is reported elsewhere (16). The present study was conducted according to the principles of the Declaration of Helsinki (59th WMA General Assembly, Seoul, October 2008) and in accordance with the local medical research regulations. The study protocol has been presented to the local Medical Ethics Committee (MEC-2017-321). No ethical approval was deemed necessary and the requirement for additional informed consent was waived.

### Neuropsychological Assessments

One of the tests in the neuropsychological assessment is the AVLT, which calls for various aspects of learning and recall. The delayed recall condition (AVLT-dr) requires patients to memorize a list of 15 words for five consecutive tests, and to recall these 15 words after 20 min. The maximal score is 15 out of 15. This test is conceptually identical to the delayed recall condition in the Wechsler Memory Scale 3—word lists used by Gondi et al. as the primary outcome measure.

In contrast to the original paper by Gondi et al., EORTC22033-26033 does not include a control group of healthy volunteers. Normal data for AVLT-dr, with test-retest changes, has been published by the Maastricht Aging Study group (17). This study tested healthy volunteers using several neuropsychological tests at 2.5 year intervals and gives parameters for a regression-based change analysis of test-retest performance. The following relationship between age and change in AVLT-dr retest score was found.

(1) $$\begin{array}{r} E=1.025-0.035\;*\;\left(age-62.5\right) \end{array}$$

Where *E* is the expected change between test and retest-score. This can be converted to a Z score using the standardized residual (which was found to be 2.362 in this test condition).

(2) $$\begin{array}{r} Z=\frac{O-E}{2.362} \end{array}$$

Where *O* is the observed retest score, and *E* is the predicted retest score. As reported in the paper by Gondi et al., a neurocognitive event was defined as a reduction in AVLT-dr score at 18 months corresponding to a Z score lower than −1.5.

### Radiotherapy Treatment

Patients were treated with photon radiotherapy using 3D conformal radiotherapy (3DCT), fractionated stereotactic radiotherapy (FSRT) or intensity modulated radiotherapy (IMRT) techniques depending on the availability at the institution. Gross tumor volume (GTV) was defined by the region of high signal intensity on T2 weighted MRI of FLAIR sequences, or, in case of prior surgery, the resection cavity and the residual tumor. Clinical target volume (CTV) margin was 10–15 mm. Planning target volume (PTV) margin was 7 mm for all patients. As required per protocol the contralateral hemisphere was spared, but no specific attempt at sparing one or both hippocampi was made.

### Delineation and DVH Analysis

A rigid registration was applied between the planning CT and MRI using MIMSoftware (Cleveland, OH, USA). Hippocampus delineation followed the instructions of the publicly available atlas from RTOG0933 (18). In case no registration was possible, delineation was performed on CT using anatomical landmarks visible on MRI. Dose volume histograms (DVH) and subsequent DVH parameters were generated for left and right hippocampus individually and for composite bilateral hippocampi. As presented in the paper by Gondi et al., we assumed an α/β value of 2 to convert physical dose to biologically equivalent dose in 2 Gy fractions (EQD2 Gy). The D*x*% of bilateral hippocampus was defined as the dose in EQD2 Gy received by *x* % of bilateral hippocampal volume.

### Statistical Approach

Descriptive statistics were generated for age, tumor laterality, tumor lobe, anti-epileptic drug treatment (AED), education, CTV volume, and hippocampal dosimetry (Table 1). The model used by Gondi et al. is based on the Lyman model (19). Their formulation was presented as follows:

(3) $$P_{NTCP}\;=\;\frac{1}{\sqrt{2\pi }}\int_{-\infty }^{t}e^{\frac{-u^{2}}{2}}du$$

Where *t* is a function of TD_50_, the dose to 40% of hippocampus at which the probability of neurocognitive decline is 50%, and *m*, is a slope parameter (see below).

(4) $$\begin{array}{r} t=\frac{D-TD_{50}}{m\;TD_{50}} \end{array}$$

In the paper published by Gondi et al., the obtained values of TD_50_ and m were 14.88 and 0.54, respectively. We applied this model to generate predicted NTCP values for the dose distributions in our study population. Cases were grouped in three bins of equal size, according to ascending NTCP. In order to compute the observed risk the incidence of a neuropsychological event in each bin is computed. The predicted NTCP was plotted against observed NTCP in a calibration plot. Next, a linear regression was performed. The regression coefficients can be used to calibrate the model to the dataset, the constant can be used as offset parameter and the slope indicates over- or underestimation of the observed risk.

**Table 1:** Patient characteristics.

**Age (years)**	**43.0**	**(95% CI 27.8–69.4)**	
Sex	Male	18	62.1%
	Female	11	37.9%
Handedness	Right	24	82.8%
	Left	5	17.2%
Years of education	13.8	(95% CI 12.0–14.4)	
Hemisphere	Right	10	34.5%
	Left	16	55.2%
	Both	3	10.3%
Lobe	Frontal	10	34.5%
	Temporal	6	20.7%
	Parietal	2	6.9%
	Multifocal	10	34.5%
	Other	1	3.4%
CTV volume (cc)	337	(95% CI 278–403)	
Number of AEDs	0	3	10.3%
	1	2	6.9%
	2	24	82.8%
Epilepsy	No seizures	9	31.0%
	Generalized tonic-clonic seizures	4	13.8%
	Partial seizures	12	41.4%
	Other	4	13.8%
Technique	3DCT	23	79.3%
	IMRT	3	10.3%
	FSRT	3	10.3%
Resection status	Biopsy	15	51.7%
	Partial removal	12	41.4%
	Total removal	2	6.9%
IDH mutation	Present	27	98.1%
	Absent	1	3.4%
	Undetermined	1	3.4%
1p/19q codeletion	Present	10	34.4%
	Absent	14	48.3%
	Undetermined	5	17.2%

*For age, CTV volume, and years of education the mean is reported along with the 95% confidence interval*.

In order to quantify model performance, the Brier score (*BS*) was calculated for the original formulation of the model. *BS* is a measure of the accuracy of a prediction with a binary outcome:

(5) $$BS\;=\;\frac{1}{n}\sum_{a=1}^{n}(f_{a}-\;o_{a}{)}^{2}$$

Where *n* is the number of observations, *f*_*a*_ is the probability that was forecast, and *o*_*a*_ is the outcome (1 if the event occurs and 0 if it does not occur). A low Brier score is indicative of good model performance, it reflects a strong correlation between forecast and outcome.

### Other Predictive Parameters

In addition to evaluating the performance of the NTCP model, we investigated if CTV volume, laterality, age, handedness, and WHO performance score were associated with cognitive deterioration. To this end, using Spearman's correlation coefficient, a correlation matrix was made to identify if bilateral and contralateral hippocampal DVH parameters correlated with cognitive deterioration.

### Power Considerations

In the paper by Gondi et al., a lower rate of neurocognitive impairment was found in the group of patients with a low dose to bilateral hippocampi, defined as dose to 40% of bilateral hippocampus volume in EQD2 Gy (D40%BH) ≤7.3 Gy (11.1 vs. 66.7%). In order to detect this difference in our group with 80% power and 2-sided significance level α = 0.05, using a Fischer exact test, and assuming the low-dose and the high-dose group are equally sized, 15 patients are required per group. The power calculation was done in SAS software version 14.1, all other statistics were done in IBM SPSS Version 24 except for the Brier score, which was calculated in MATLAB v2017a.

## Results

### Patient Data

**Figure 1:**
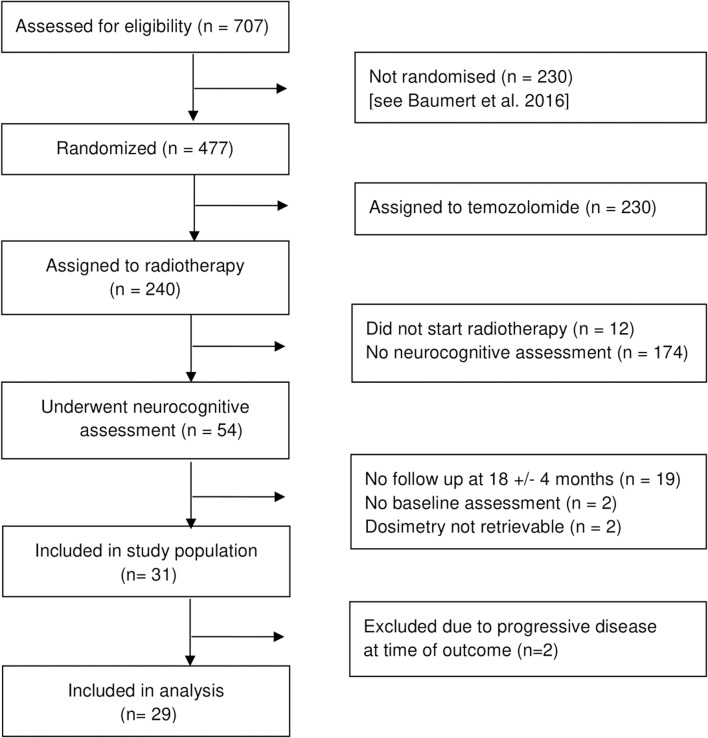
Inclusion of patients.

Of 477 patients within EORTC 22033-26033, 103 patients underwent full neurocognitive testing. Of these, 54 patients were treated with radiotherapy-only. Of these, 33 patients had a complete neurocognitive assessment at baseline and at a median follow up of 18.5 months (95% CI 17.3–18.9). Complete original dosimetry data was available for 31 patients. Two patients were excluded due to clinically progressive disease at time of neurocognitive outcome assessment (Figure 1).

**Figure 2:**
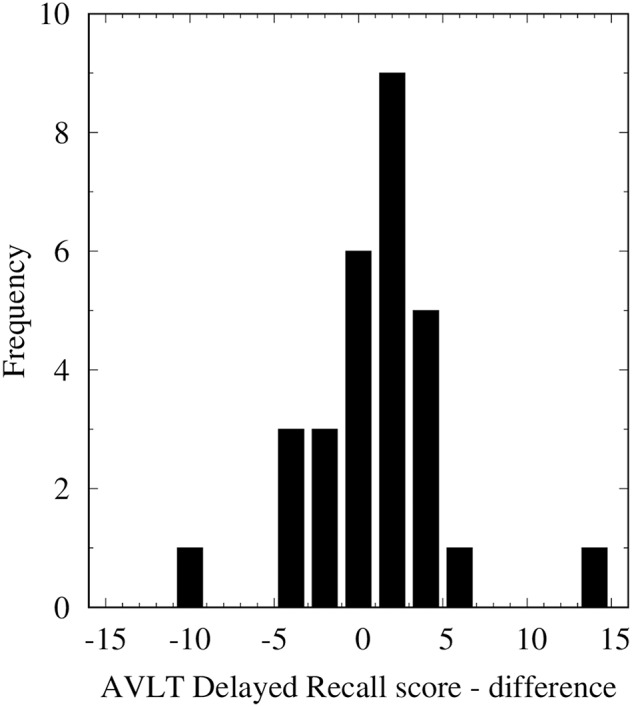
Histogram of differences in AVLT-dr score per patient (baseline minus follow-up). Overall, there was no significant difference between pre- and post-radiotherapy AVLT-dr score.

Data of 29 patients from 1 Spanish and 4 Dutch institutes is summarized in Table 1. Median age of patients at randomization was 43 years (95% CI 39–47). Only three patients did not require anti-epileptic medication. Sixteen tumors were left sided, 10 right sided, and three were bilateral. Final resection status was biopsy only in 15 patients, gross total resection in two patients, and partial resection in twelve patients. An IDH mutation was present in 27 patients, absent in one patient and undetermined in one patient. An 1p/19q codeletion was present in 10 patients, absent in 14 patients, and undetermined in five patients. Twenty-eight patients were treated to a dose of 50.4 Gy in 28 fractions, one patient was treated to a dose of 54 Gy in 30 fractions. Twenty-five patients were treated with 3DCT, three with IMRT and two with fractionated stereotactic radiotherapy. Mean CTV volume was 340 cc (95% CI 276–403). Mean dose in EQD2 Gy to bilateral hippocampi was 31.4 Gy (95% CI 27.2–35.6). The mean D40%BH was 40.9 Gy (95% CI 35.8–46.0), and the median D40%BH was 47.2 Gy. Only one patient had a D40%BH lower than 7.3 Gy. Mean dose in EQD2 Gy to contralateral hippocampus was 21.6 Gy (95% CI 16.7–26.9). Overall, there was no significant difference between pre- and post-radiotherapy AVLT-dr score (95% CI 1.09–2.16; Figure 2). A cognitive event was scored in seven patients (24.1%). At the time of analysis, the median time to progression in 14 patients was 2.9 years (95% CI 2.2–3.6). Fifteen patients were free of progressive disease after a median follow-up duration of 3.3 years. We compared the subgroup of patients with available data (*n* = 31) with the rest of the study population (*n* = 446). The groups were comparable with respect to tumor laterality, tumor lobe, performance status, progression free survival, and presence of an 1p/19q codeletion. However, the number of IDH wildtype tumors was significantly lower in the study population (3.2 vs. 14%, *p* = 0.025, see Supplementary Data).

### Model Performance

**Figure 3:**
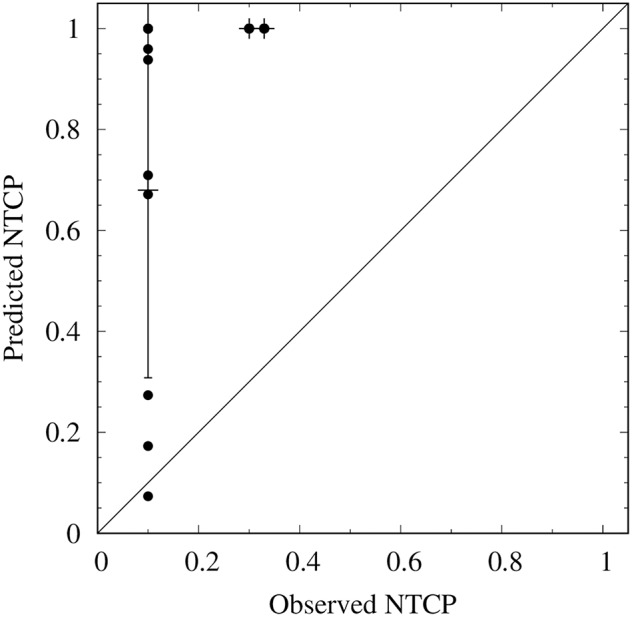
Calibration plot of the original model in this dataset. The predicted NTCP is calculated using the NTCP model. The observed NTCP is calculated by sorting the cases in three bins of ascending risk (horizontal axis), and computing the incidence of a neuropsychological event in each bin (vertical axis). The intercept line represents agreement between predicted and observed NTCP. Error bars are 95% confidence intervals.

We were unable to compare the incidence of cognitive events between the high and low dose group as described in the paper by Gondi et al. (D40%BH <7.3 Gy) as there was only one case in the low dose group. However, there was no difference in the incidence of a cognitive event between the group that received a D40%BH above vs. below the median (47.2 Gy) in this study (14 vs. 25%, *p* = 0.68). NTCP values are presented in Table 2 with dosimetry and neurocognitive results. A calibration plot is presented in Figure 3. Linear regression showed a constant of 0.03 (*p* = 0.60) and a slope of 0.24 (*p* < 0.01) at an *r*^2^ of 0.346. The Brier score of the model was 0.63.

**Table 2:** Dosimetric parameters, expected values derived from the NTCP model, and cognitive event (see text for definition).

**Age**	**Dose to 40% of bilateral hippocampus (EQD2 Gy)**	**Predicted NTCP (NTCP model)**	**Cognitive event**
48.7	3.21	0.07	No
48.0	7.30	0.17	No
36.3	10.04	0.27	No
69.4	18.45	0.67	Yes
49.2	19.31	0.71	No
45.5	27.24	0.94	No
42.3	28.89	0.96	No
40.3	40.62	>0.99	No
32.9	44.30	>0.99	No
50.8	45.51	>0.99	No
37.1	46.27	>0.99	Yes
35.6	46.52	>0.99	No
40.6	46.79	>0.99	No
41.6	47.08	>0.99	No
50.1	47.18	>0.99	Yes
34.7	47.31	>0.99	No
48.5	47.42	>0.99	No
60.2	47.50	>0.99	Yes
35.9	47.61	>0.99	No
36.3	47.87	>0.99	No
42.5	47.91	>0.99	No
29.5	47.91	>0.99	No
35.2	48.00	>0.99	No
66.5	48.13	>0.99	No
44.3	48.20	>0.99	Yes
34.0	48.43	>0.99	No
50.6	48.73	>0.99	Yes
32.0	48.95	>0.99	No
27.8	50.74	>0.99	No

### Dosimetric Parameters

**Figure 4:**
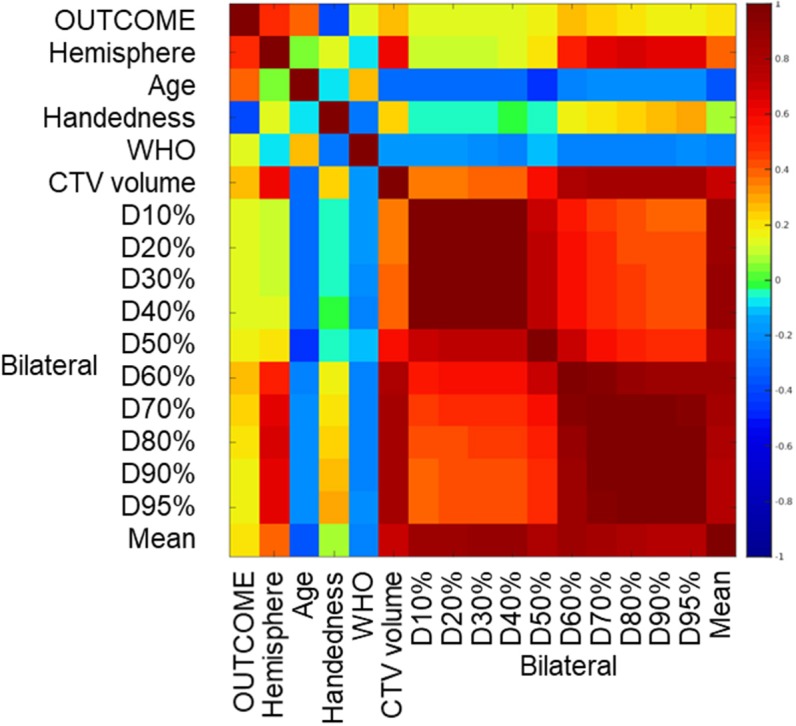
Correlation matrix of clinical and dosimetric parameters. Outcome: neurocognitive event, binary (for definition, see “Methods”), WHO, World Health Organization Performance score; D10%, 20%, etc., dose absorbed by 10, 20%, etc. of bilateral hippocampus volume. Color: Spearman correlation coefficient. There are significant correlations between age and outcome, laterality and outcome, and between individual dosimetric parameters.

A heat map of the correlation matrix is presented in Figure 4. Increasing age (*p* = 0.04) and tumor localization in the left hemisphere (*p* = 0.01) were related to poorer neurocognitive outcome at 18 (±4) months. None of the bilateral hippocampal dose volume parameters (D10%, D20%, D30% up to D90%, D95% and mean dose) did exhibit a significant correlation with outcome.

## Discussion

To the best of our knowledge, this is the first attempt to quantify the performance of the hippocampal NTCP model within a group of only LGG patients treated with partial brain irradiation. This model was used in RTOG 0933—hippocampal sparing whole brain radiotherapy vs. standard whole brain therapy in brain metastases and in the recently presented phase III trial exploring WBRT plus memantine, with or without hippocampal avoidance (NRG-CC001) (18, 20). Brain metastases are almost never observed in the hippocampus, and selective avoidance of this region is not likely to result in a higher risk of intracranial recurrence (21). This is less clear in LGG where tumor cells are known to be present within the entire brain (22). Moreover, subventricular zone involvement has been shown to be a biomarker for poor prognosis (23), making the hippocampus a potential treatment target.

In the calibration procedure, the positive slope in the linear regression indicates an overestimation of NTCP values by the model in this dataset. The high Brier score indicates poor model performance. In comparing the two study groups, the incidence of a neurocognitive event is similar (29.2 vs. 24.1% in this study) but the range of hippocampal dose is quite different. The median D40%BH in the paper by Gondi et al. was 7.3 Gy, at above which a NTCP of 66.7% was observed. By contrast, the median D40%BH in this paper is 47.2 Gy and all but one of the patients in the present study received a D40%BH in excess of 7.3 Gy. In comparing the two groups, there are substantial differences in the delivery technique and target volume. In the paper by Gondi et al., most patients were treated without a CTV expansion and with limited PTV margins (2 mm) using highly conformal dose distributions. In the present study, patients were treated with a CTV margin of 10–15 mm and a larger PTV (7 mm) resulting in substantially larger target volumes, and the delivery technique was mainly 3DCRT. It is likely that this resulted in higher doses to bilateral hippocampus in this study, to a degree that almost none of the patients were in the low dose group. As such, we were unable to compare the incidence of neurocognitive impairment between the high dose and the low dose group. However, the hippocampal doses in this study group are probably a good representation of the hippocampal dose range found in LGG patients undergoing radiotherapy. Therefore, this study should not be read as a formal disapproval of the hippocampal NTCP model, but rather as a caution toward extrapolating a NTCP model beyond the dose range in which it was developed. A similar issue was encountered by Moiseenko in comparing NTCP models for radiation toxicity to the visual apparatus (24). Since no significant correlation between dosimetric parameters and outcome was observed, we were unable to generate an alternative model from this dataset.

The choice of endpoint, neurocognitive failure at 18 months after radiotherapy, is debatable in LGG patients. Trials that found a significant effect of radiotherapy on neurocognitive function typically only did so after a follow-up >5 years (7, 8), whereas several trials with a shorter follow-up found no significant, or only transient, deleterious effects (9–12, 25). This begs the question whether neurocognitive impairment at 18 months is indeed indicative of a persistent neurocognitive deficit.

Although preclinical and radiological (26, 27) data demonstrated appreciable changes within the hippocampus after radiotherapy, a relationship between cognitive performance and a D40% as low as 7.3 EQD2 Gy was not found in the current study but also not in other studies. In the setting of prophylactic WBRT in small cell lung cancer and partial brain irradiation for glioblastoma multiforme, Ma et al. (28) demonstrated D50% of 22.1 Gy to be associated with a 20% risk of a significant decline in Hopkins Verbal Learning Test (HVLT)—delayed recall score. Peiffer et al. (29) identified the volume of bilateral hippocampi receiving 60 Gy as a possible predictor for cognitive decline. The analysis by Okoukoni et al. (30) established a correlation between post-treatment HVLT score (no baseline measurement was done) and even higher doses to the bilateral hippocampi. Here, hippocampal V55 Gy of 0, 25, and 50% were associated with post-radiation impairment rates of 14.9, 45.9, and 80.6%, respectively.

In this study, we used prospectively acquired baseline and follow up data from the recently completed EORTC22033-26033 trial, ensuring a homogenous patient group with good adherence to protocol. The subset of patients included in this analysis is a relatively small proportion of the radiotherapy-only group (15%). The main reason for this is that neurocognitive testing was not mandatory, and a number of centers did no neurocognitive testing. However, we found no significant differences in clinical variables (save for presence of IDH mutation) and time to progressive disease between our subset of and rest of the study population. In comparing our neurocognitive event-definition to the one used in the paper by Gondi et al., we did not utilize a control group but published test-retest data from the Maastricht Aging study. This data is derived from a study group that is older (49–56 years), than the average patient in our study (43 years), and the test-retest interval is twice as long (3 years).

In this dataset of only LGG patients, the NTCP model did not perform as expected in predicting cognitive decline based on dose to bilateral hippocampus. Clearly, the understanding of the relationship between dose to subsites in the CNS and neurocognitive functioning is still limited, and there exists a paucity of prospective neuropsychological and dosimetric parameters with an adequate duration of follow-up.

## Data Availability Statement

The datasets generated for this study are available on request to the corresponding author.

## Ethics Statement

This study was carried out in accordance with the Dutch Medical Research (Human Subjects) Act (WMO). The protocol was approved by the Medical Research Ethics Committee Erasmus MC. All subjects gave written informed consent in accordance with the Declaration of Helsinki.

## Author Contributions

The study was conceptualized and the manuscript was written by JJ and AM. The statistical analysis was done by JJ and MH. MB, MT, and MK helped interpreting the data. JJ, RW, DE, FL, and AL were involved in data collection. All authors critically reviewed the manuscript and commented on the final version. The final authorship position is shared between BB and MK.

### Conflict of Interest

The authors declare that the research was conducted in the absence of any commercial or financial relationships that could be construed as a potential conflict of interest.
