# Supplementary material for: Evaluation of the Hippocampal Normal Tissue Complication Model in a Prospective Cohort of Low Grade Glioma Patients—An Analysis Within the EORTC 22033 Clinical Trial
Source: Front Oncol. 2019 Oct 1;9:991. doi: 10.3389/fonc.2019.00991 (PMC6797857; doi:10.3389/fonc.2019.00991)
Supplement: Supplementary file 1 [file Table_1.DOCX]

| **Comparison with other RT patients** | | |  |
| --- | --- | --- | --- |
|  |  | |  |
|  | **EORTC22033 (N=209)** | **This study (N=31)** | **P-value** |
|  | **N (%)** | **N (%)** |  |
| **Hemisphere** |  |  |  |
| **Right** | 90 (43.1) | 10 (32.3) | 0.24* |
| **Left** | 106 (50.7) | 18 (58.1) |  |
| **Both** | 9 (4.3) | 3 (9.7) |  |
| **Missing** | 4 (1.9) | 0 (0.0) |  |
| **Lobe** |  |  |  |
| **Frontal** | 80 (38.3) | 11 (35.5) | 0.67* |
| **Temporal** | 42 (20.1) | 6 (19.4) |  |
| **Parietal** | 16 (7.7) | 2 (6.5) |  |
| **Occipital** | 2 (1.0) | 0 (0.0) |  |
| **Basal ganglia** | 1 (0.5) | 0 (0.0) |  |
| **Multifocal** | 44 (21.1) | 11 (35.5) |  |
| **Other** | 20 (9.6) | 1 (3.2) |  |
| **Missing** | 4 (1.9) | 0 (0.0) |  |
| **Performance Status** |  |  |  |
| **0** | 129 (61.7) | 22 (71.0) | 0.33** |
| **1** | 71 (34.0) | 8 (25.8) |  |
| **2** | 9 (4.3) | 1 (3.2) |  |
| **IDH status** |  |  |  |
| **mutaded** | 136 (65.1) | 28 (90.3) | 0.02* |
| **normal** | 34 (16.3) | 1 (3.2) |  |
| **undetermined** | 39 (18.7) | 2 (6.5) |  |
| **1p/19q deletion** |  |  |  |
| **1p/19q codeleted** | 45 (21.5) | 10 (32.3) | 0.40* |
| **1p/19q no codeleted** | 110 (52.6) | 15 (48.4) |  |
| **Undetermined/missing** | 54 (25.8) | 6 (19.4) |  |

Note: * Fisher test, ** Wilcoxon rank sum test

Supplementary data 1: A comparison of descriptive statistics for the study cohort (n=31) and the EORTC22033-26033 radiotherapy group

| **Progression Free Survival** | | | **Non-parametric** | | **Cox model** | |
| --- | --- | --- | --- | --- | --- | --- |
| **In the subgroup** | **Patients (N)** | **Observed Events (O)** | **Median (95% CI) (Months)** | **% at 3 Year(s) (95% CI)** | **Hazard Ratio (95% CI)** |  |
| **EORTC22033** | 209 | 110 | 45.86 (35.52, 55.13) | 56.8 (49.4, 63.6) | 1.00 |  |
| **This study** | 31 | 16 | 50.99 (40.67, N) | 71.0 (51.6, 83.7) | 0.85 (0.50, 1.44) |  |
|  |  |  |  |  | Log-rank test: | p-value=0.539 |

| **Comparison with other patients** | | |  |
| --- | --- | --- | --- |
|  |  | |  |
|  | **EORTC22033 (N=446)** | **This study (N=31)** | **p-value** |
|  | **N (%)** | **N (%)** |  |
| **Hemisphere** |  |  |  |
| **Right** | 199 (44.6) | 10 (32.3) | 0.21* |
| **Left** | 221 (49.6) | 18 (58.1) |  |
| **Both** | 22 (4.9) | 3 (9.7) |  |
| **Missing** | 4 (0.9) | 0 (0.0) |  |
| **Lobe** |  |  |  |
| **Frontal** | 185 (41.5) | 11 (35.5) | 0.68* |
| **Temporal** | 84 (18.8) | 6 (19.4) |  |
| **Parietal** | 32 (7.2) | 2 (6.5) |  |
| **Occipital** | 2 (0.4) | 0 (0.0) |  |
| **Brain Stem** | 1 (0.2) | 0 (0.0) |  |
| **Basal ganglia** | 1 (0.2) | 0 (0.0) |  |
| **Multifocal** | 100 (22.4) | 11 (35.5) |  |
| **Other** | 37 (8.3) | 1 (3.2) |  |
| **Missing** | 4 (0.9) | 0 (0.0) |  |
| **Performance Status** |  |  |  |
| **0** | 272 (61.0) | 22 (71.0) | 0.28** |
| **1** | 157 (35.2) | 8 (25.8) |  |
| **2** | 17 (3.8) | 1 (3.2) |  |
| **IDH status** |  |  |  |
| **mutaded** | 299 (67.0) | 28 (90.3) | 0.025* |
| **normal** | 64 (14.3) | 1 (3.2) |  |
| **undetermined** | 83 (18.6) | 2 (6.5) |  |
| **1p/19q deletion** |  |  |  |
| **1p/19q codeleted** | 107 (24.0) | 10 (32.3) | 0.58* |
| **1p/19q no codeleted** | 225 (50.4) | 15 (48.4) |  |
| **Undetermined/missing** | 114 (25.6) | 6 (19.4) |  |

Note: * Fisher test, ** Wilcoxon rank sum test

Supplementary data 2: A comparison of descriptive statistics for the study cohort (n=31) and the entire EORTC22033-26033 study population

| **Progression Free Survival** | | | **Non-parametric** | | **Cox model** | |
| --- | --- | --- | --- | --- | --- | --- |
| **In the subgroup** | **Patients (N)** | **Observed Events (O)** | **Median (95% CI) (Months)** | **% at 3 Year(s) (95% CI)** | **Hazard Ratio (95% CI)** |  |
| EORTC22033 | 446 | 246 | 41.13 (35.35, 46.46) | 54.4 (49.4, 59.1) | 1.00 |  |
| This study | 31 | 16 | 50.99 (40.67, N) | 71.0 (51.6, 83.7) | 0.78 (0.47, 1.29) |  |
|  |  |  |  |  | Log-rank test: | p-value=0.325 |
